# Phage Small Proteins Play Large Roles in Phage-Bacterial Interactions

**DOI:** 10.1016/j.mib.2024.102519

**Published:** 2024-07-22

**Authors:** Grace A. Beggs, Bonnie L. Bassler

**Affiliations:** 1 Department of Molecular Biology, Princeton University, Princeton, NJ 08544, USA; 2 Howard Hughes Medical Institute, Chevy Chase, MD 20815, USA

## Abstract

Phages have wide influence on bacterial physiology, and likewise, bacterial processes impinge on phage biology. Key to these interactions are phage small proteins (<100 aa). Long underappreciated, recent work has revealed millions of phage small proteins, and increasingly, mechanisms by which they function to dictate phage and/or bacterial behavior and evolution. Here, we describe select phage small proteins that mediate phage-bacterial interactions by modulating phage lifestyle decision-making components or by altering host gene expression.

## Introduction

Foundational studies of bacteriophages (phages), viruses that infect bacteria, ushered in the molecular biology revolution. Now, 80 years later, a resurgence in interest in phages has revealed new and unexpected phage biology and phage-bacterial interactions [1]. Metagenomic studies have uncovered the diversity of phage genome architectures and distributions across ecosystems (reviewed in [2,3]). Molecular analyses have defined mechanisms underlying bacterial anti-phage defense systems, phage anti-defense systems, and phage-phage and phage-bacterial chemical signaling. Many of these newly revealed phage processes rely on components with unknown functions. Germane to this piece are the discoveries of over 2 million small genes in phage genomes that frequently encode proteins of unknown function [4,5]. In studied cases, these phage small proteins clearly play central roles in phage and bacterial biology, potentially including in the human microbiome [4,5]. Here, we focus on recent revelations of molecular mechanisms underlying the functions of novel phage-encoded small proteins (for our purposes, arbitrarily defined as <100 amino acids) that influence phage lifestyle decision making or affect host bacterial gene expression. We do not cover phage small proteins that primarily function in anti-defense, such as anti-CRISPRs, as these have been reviewed elsewhere [6,7]. Important functions of small proteins encoded in bacterial genomes have also been recently reviewed [8,9], so those proteins are not a subject of the current report.

## Phage Small Proteins That Direct Phage Lysogeny-Lysis Decision Making

Temperate phages can adopt one of two lifestyles. First is the lytic lifestyle, in which the phage replicates, kills its current host, escapes, and spreads by infecting neighboring cells. The second lifestyle is called lysogeny in which the phage genome is replicated along with the host genome and the phage is passed down to bacterial progeny as a lysogen. Phage-encoded small proteins often play key roles in directing transitions between these two phage lifestyles [10–12]. In the classic case of lambda-like phages, the phage-encoded regulator cI binds the P_R_ promoter to repress the phage lytic genes [13]. cI activity maintains lysogeny: following establishment of lysogeny, cI must be inactivated to enable lytic gene expression and the transition from lysogeny to lysis. The textbook model for cI inactivation is cleavage of the cI protein via a RecA-dependent mechanism in response to DNA damage [13].

**Figure 1.**
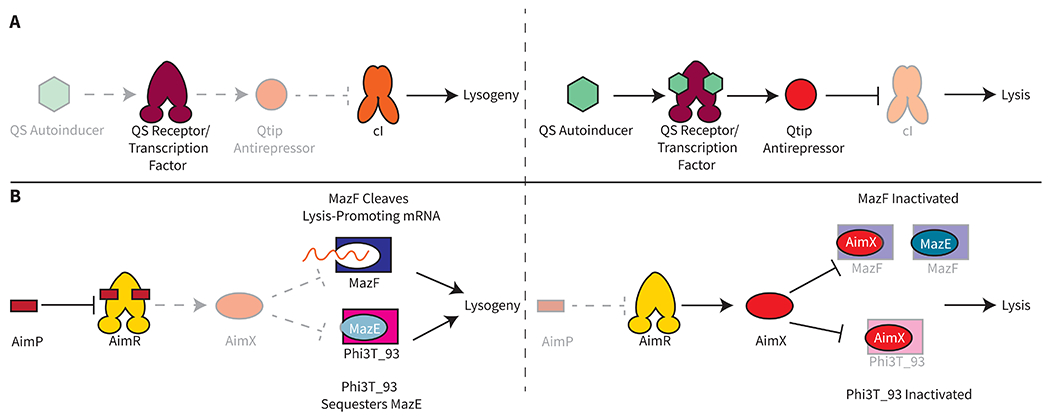
Phage Small Proteins Direct Lysogeny-Lysis Transitions. **(A)** (Left) In the absence of the quorum-sensing (QS) autoinducer, cI from phage VP882 functions to maintain lysogeny. (Right) When the QS autoinducer is present, it binds to a phage encoded QS receptor/transcription factor that activates expression of the *qtip* gene encoding the Qtip antirepressor. Qtip, in turn, inactivates cI by sequestration, enabling lytic gene expression. **(B)** (Left) In phage φ3T, the AimP peptide binds to the AimR transcription factor to repress its DNA-binding activity. As a consequence, the host factor MazF cleaves RNA transcripts encoding phage lytic genes. Additionally, the phage protein Phi3T_93 sequesters the MazE antitoxin, which enables MazF RNase activity. Both of these activities lead to lysogeny. (Right) In the absence of AimP, AimR activates expression of *aimX*, and AimX binds to Phi3T_93 and to MazF which suppresses MazF RNase activity, leading to phage-driven lysis.

Upending this traditional view are recent studies showing that phage small proteins can drive transitions from lysogeny to lysis by inactivating cI [12,14–16]. Some of these phage small proteins are antirepressors that inactivate their target cI-type repressors in response to DNA damage or, alternatively, in response to external cues such as quorum-sensing (QS) signals. QS involves the production, release, and group-wide detection of signal molecules called autoinducers [11]. Bacteria use QS to orchestrate collective behaviors. QS-responsive phage antirepressors have been discovered in vibriophage VP882 and *Aeromonas* phages [12,14,15]. Phages possessing QS-controlled antirepressors are capable of transitioning from lysogeny to lysis exclusively at high host cell density. This cunning strategy presumably maximizes transmission of phages to new host cells. The first discovered QS-controlled phage antirepressor is called Qtip, a 79 amino acid protein from phage VP882 [14,15]. Expression of *qtip* is activated at high cell density by a phage encoded QS receptor/transcription factor that detects the accumulation of its host bacterium’s QS autoinducer. Qtip sequesters cI, driving the transition from lysogeny to lysis (Figure 1A). Database analyses reveal many phages harboring similar two-gene modules specifying a transcription factor and partner small protein [12,14]. Little to no homology exists among these phage small proteins. However, in systems that have been studied, the phage small proteins are antirepressors that inactivate their cognate cI repressors.

Beyond driving lysis, phage small proteins play roles in maintaining lysogeny. Take *Escherichia* phage P1; it encodes two small proteins, Coi an antirepressor that drives lysis, and Lxc a co-repressor, that functions with cI to maintain lysogeny [17]. A particularly curious example is the phage-encoded modulator of repression (MOR) from the *Lactococcus lactis* phage TP901-1. MOR is a 72 amino acid protein containing a helix-turn-helix motif. MOR inhibits cI-operator binding by blocking the O_L_-binding site. Thus, MOR drives the transition from lysogeny to lysis [18]. However, MOR has a dual role as it also acts as a co-repressor with cI to keep P_R_ repressed and maintain lysogeny. A few models have been proposed to suggest how MOR may undertake its two opposing roles [18–20]. For example, it has been suggested that MOR alters the binding affinity of the cI multimer, specifically to prevent cI-O_L_ binding while maintaining repression at P_R_ [18,19]. An in-depth understanding of the MOR mechanism remains to be established and is an important open question considering the high conservation of MOR across a range of phages.

Temperate phages that do not harbor classic lambda-like components, such as the SPβ phages, often use small proteins to direct lifestyle decision making via mechanisms that may or may not involve a repressor that functions analogously to cI [21–23]. A remarkable example is the arbitrium system, which relies on QS-signaling between prophages residing in *Bacillus* [21,24,25]. Various *Bacillus* prophages produce and secrete a peptide called AimP. AimP binds to the phage-encoded AimR receptor, and the complex promotes lysogeny. In the case of prophage φ3T, when AimP accumulates and binds AimR, the AimP-AimR complex is unable to bind DNA and activate transcription of the phage *aimX* gene encoding the small protein AimX (51 amino acids) [26,27]. Under this condition, the lysogeny-promoting factor, Phi3T_93 sequesters the host MazE antitoxin, which, in turn, frees the MazF toxin to cleave transcripts encoding phage lytic genes, promoting lysogeny (Figure 1B). In the absence of AimP, AimR binds DNA and activates expression of *aimX*. AimX binds MazF to inhibit its RNase activity. AimX also keeps Phi3T_93 from sequestering the MazE antitoxin. The consequence is lysis. The spectacular feature of the arbitrium system is that only when many hosts are infected with prophages, can the secreted AimP accumulate to the threshold required for detection. This regulatory arrangement ensures that lysis only occurs when most of the host bacterial population consists of naïve cells that are vulnerable to infection. Recent mechanistic insight into how this system functions highlights that phage small proteins can act via interaction with phage or host-encoded factors. We note that small proteins encoded by lytic phages that are incapable of lysogeny can also drive lysis through interaction with host factors [28,29]. These small proteins have been reviewed elsewhere [30].

## Phage Small Proteins That Alter Host Gene Expression

Phage small proteins can alter host gene expression by interacting with host proteins. Indeed, recent data show that phage small proteins can reprogram the host transcriptome by interaction with bacterial RNA polymerase (RNAP) [31,32]. Many phages encode homologs of TraR, a 73 amino acid protein first identified in the *Escherichia coli* F conjugation plasmid. In *E. coli*, TraR binds RNAP and in so doing mimics the DksA-ppGpp-RNAP binding that occurs during the stringent response [32]. While F plasmid TraR production/activity is not known to be induced by the stringent response [32,33], TraR regulates the expression of many of the same promoters as do DksA/ppGpp including those encoding ribosome components and amino acid biosynthesis enzymes, potentially regulating cell growth and membrane repair during conjugation. In addition to TraR from the F plasmid, other phage-encoded TraR homologs bind RNAP [32]. Characterization of the TraR homolog encoded on phage Lambda, called λ Orf73, revealed that λ Orf73 binds RNAP to inhibit transcription from rRNA and ribosomal protein promotors. Intriguingly, λ Orf73 does not affect transcription of phage lytic genes but does increase phage yield through its regulation of host promoters [32]. The notion is that λ Orf73 functions to slow host cell growth, perhaps preventing production of new host translation machinery. Slowed host growth is presumed to buy the phage extra time between cell divisions to increase virion production.

**Figure 2.**
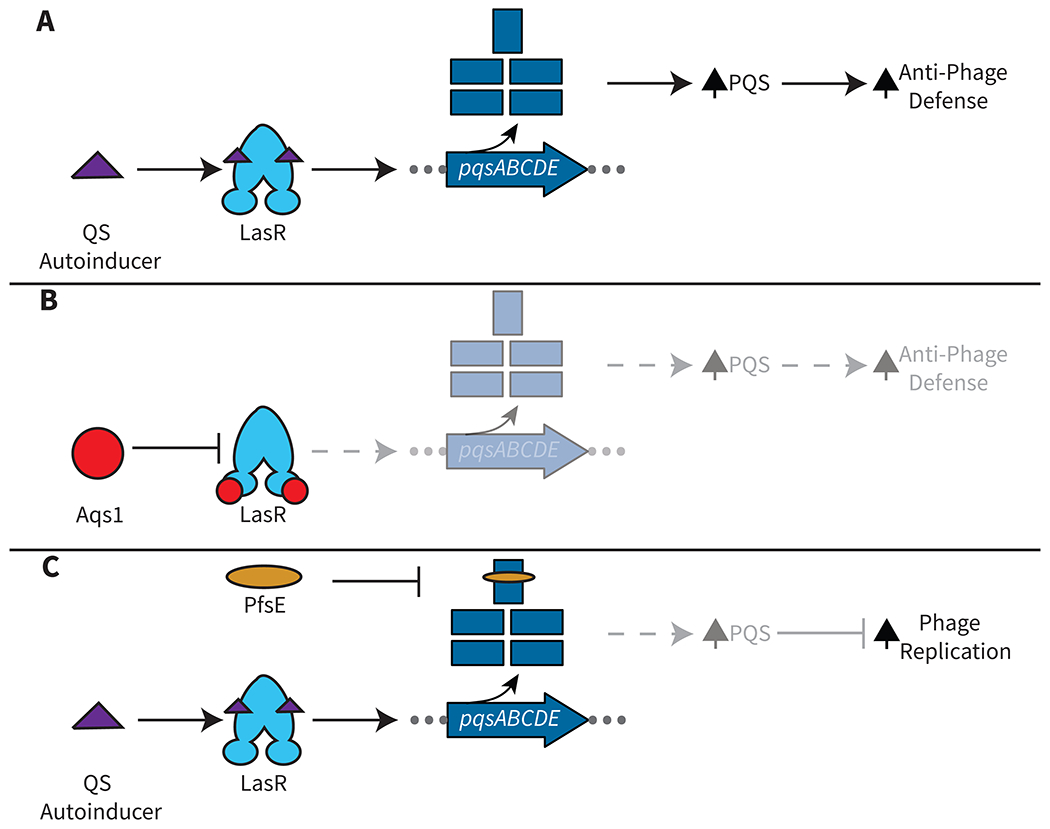
Phage Small Proteins Inhibit Host Quorum Sensing (QS). **(A)** In the presence of autoinducer, *P. aeruginosa* LasR upregulates the PQS biosynthetic pathway, which, in turn, promotes activation of genes encoding anti-phage defense systems. The consequence is protection of the bacterial population against phage infection. **(B)** The *Pseudomonas* phage small protein Aqs1 binds to and inhibits host LasR activity, which decreases PQS production and prevents upregulation of anti-phage defense systems, protecting the phage. **(C)** The *Pseudomonas* phage small protein PfsE binds to and inhibits host PqsA to block PQS production and enable enhanced phage virion production.

Another strategy by which phages use small proteins to globally affect host gene expression is by interfering with host QS. Some of the best characterized examples come from *Pseudomonas* phages, which encode proteins that target either QS transcriptional regulators or the biosynthetic enzymes responsible for producing QS autoinducers [34–37]. The 69 amino acid protein Aqs1, first characterized in phage DMS3 but present in many *Pseudomonas* phages, binds the master QS-regulator, LasR, inhibiting its activity (Figure 2A and 2B). The consequence is a reduction in production of the PQS QS autoinducer, suppression of QS-controlled anti-phage defense genes, and protection of the phage [34].

In another example from *Pseudomonas*, rather than targeting a QS transcriptional regulator, the small PfsE protein encoded by phage Pf4 binds to and inhibits the PqsA biosynthetic enzyme (Figure 2C), responsible for catalyzing the first step in PQS synthesis [35]. Disabling PQS-mediated cell-cell communication leads to increased Pf4 phage replication efficiency, perhaps by freeing up host resources. Pf phages have also been broadly associated with both activating and repressing different host QS regulators to affect *Pseudomonas* virulence, so likely there are phage factors in addition to PfsE that interact with host QS [38]. Beyond *Pseudomonas* phages, a small protein that functions analogously to Aqs1 has been discovered in a phage that infects *Streptococcus pyogenes* [39,40]. In this case, the phage protein Prx, which is highly conserved throughout Group A *Streptococcus* bacteriophages, inhibits the QS-regulator ComR, a member of the RRNPP protein family (named for the prototypical members Rap, Rgg, NprR, PlcR, and PrgX), by binding conserved residues in the ComR DNA-binding domain. Interaction of Prx with ComR inhibits *S. pyogenes* natural competence. This mechanism allows *Streptococcus* phages to block processes that occur concurrently with host transformation that are often destabilizing to the prophage, such as homologous recombination and DNA repair. Although multiple strategies for phage small proteins targeting QS have been uncovered, to date, studies have focused on how these phage small proteins affect virion production. The broader implications for global host biology remain to be investigated.

Phages do not restrict the use of their small proteins to targeting host QS. Other host processes such as the SOS response, virulence factor production or activity, and metabolic pathways are also targets [41–44]. For example, the classic lytic regulator Cro (a small helix-turn-helix motif containing protein) encoded by an Stx-lambdoid-like phage present in Enterohemorrhagic *E. coli* (EHEC) activates expression of host genes encoding the type III secretion system, a central EHEC virulence process [44]. Intriguingly, the Cro regulator of this phage displays basal activity when the phage exists as a lysogen. Thus, having this phage as a resident can confer an advantage to EHEC by enhancing its virulence capacity. Another example, Gp7 from *Bacillus* phage GIL01, is a small protein that interacts with the host SOS-regulator LexA to repress genes required for the SOS response, protein hydrolysis, and membrane transport [43]. Gp7 upregulates genes specifying a few virulence factors including pore-forming cytolysins. The ramifications to the host that are attributed to Gp7 remain somewhat unclear as other factors are hypothesized to have co-evolved with Gp7 that counteract some of its regulatory activities. We note that while not the focus of the current review, some phage small proteins thwart crucial host processes via mechanisms that do not alter gene expression, including through interaction with FtsZ to block protofilament assembly and thus inhibit cell division (i.e., Kil from phage Lambda and Gp0.4 from phage T7) [45–48], and inhibition of the Lon protease (i.e., Gp4.5 from phage T7) [49]. How suppression of cell division and protease activity benefits these phages remains to be defined.

## Conclusions

The above examples make clear the versatility of phage small proteins in modulating phage and host biology. Technological advances will continue to reveal new phage genes [50,51]. Understanding mechanisms by which phage small proteins function remains a daunting but exciting challenge given that, to date, these proteins are new and by default understudied. Database analyses can reveal whether or not homologs of a newly discovered phage small protein exist, and thus, can provide insight into broad or narrow spectrum, but that does not yield function. Despite recent spectacular developments in computational structural predictions (i.e., AlphaFold), these technologies often fail when the enquiry is a poorly conserved, short protein sequence [52]. Thus, biochemical and structural analyses together with genetic and phenotypic studies will continue to be vital to defining the mechanisms by which this treasure trove of phage small proteins affects phage and host biology. Such studies will likely yield novel mechanisms in both the phages and their hosts, and with luck, a broader understanding of small protein biology that goes beyond bacteriophages [53]. Moreover, while beyond the scope of this review, larger phage-encoded proteins have recently been shown to regulate host processes including nucleoid compaction, cell adhesion, and sporulation [54–56]. Additionally, larger phage proteins are now known to bind the ribosomal translation machinery but the outcome to the phage and to the host remain unknown [57]. In conclusion, phage small (and large) proteins represent a largely untapped and growing area worthy of study as it is already clear they can function as lynchpins modulating phage-bacterial interactions.

## Highlights

- Phage small proteins play central roles in phage and bacterial physiology.
- Phage small proteins can direct phage lifestyle decision-making.
- Phage small proteins can alter bacterial host gene expression.
- Mechanistic studies of phage small proteins are essential to define functions.
